# Informatics Methodology Used in the Web-Based Portal of the NASCITA Cohort Study: Development and Implementation Study

**DOI:** 10.2196/23087

**Published:** 2021-03-12

**Authors:** Michele Zanetti, Antonio Clavenna, Chiara Pandolfini, Claudia Pansieri, Maria Grazia Calati, Massimo Cartabia, Daniela Miglio, Maurizio Bonati

**Affiliations:** 1 Laboratory for Mother and Child Health Department of Public Health Istituto di Ricerche Farmacologiche Mario Negri IRCCS Milan Italy

**Keywords:** internet, computer systems, cohort studies, pediatricians, infant, newborn

## Abstract

**Background:**

Many diseases occurring in adults can be pinned down to early childhood and birth cohorts are the optimal means to study this connection. Birth cohorts have contributed to the understanding of many diseases and their risk factors.

**Objective:**

To improve the knowledge of the health status of Italian children early on and how it is affected by social and health determinants, we set up a longitudinal, prospective, national-level, population-based birth cohort, the NASCITA study (NAscere e creSCere in ITAlia). The main aim of this cohort is to evaluate physical, cognitive, and psychological development; health status; and health resource use in the first 6 years of life in newborns, and potential associated factors. A web-based system was set up with the aim to host the cohort; provide ongoing information to pediatricians and to families; and facilitate accurate data input, monitoring, and analysis. This article describes the informatics methodology used to set up and maintain the NASCITA cohort with its web-based platform, and provides a general description of the data on children aged over 7 months.

**Methods:**

Family pediatricians were contacted for participation in the cohort and enrolled newborns from April 2019 to July 2020 at their first well-child visit. Information collected included basic data that are part of those routinely collected by the family pediatricians, but also parental data, such as medical history, characteristics and lifestyle, and indoor and outdoor environment. A specific web portal for the NASCITA cohort study was developed and an electronic case report form for data input was created and tested. Interactive data charts, including growth curves, are being made available to pediatricians with their patients’ data. Newsletters covering the current biomedical literature on child cohorts are periodically being put up for pediatricians, and, for parents, evidence-based information on common illnesses and problems in children.

**Results:**

The entire cohort population consists of 5166 children, with 139 participating pediatricians, distributed throughout Italy. The number of children enrolled per pediatrician ranged from 1 to 100. The 5166 enrolled children represent 66.55% (5166/7763) of the children born in all of 2018 covered by the same pediatricians participating in the cohort. The number of children aged over 7 months at the time of these analyses, and for whom the most complete data were available upon initial analyses, was 4386 (2226/4381 males [50.81%] and 142/4370 twins [3.25%]). The age of the mothers at birth of the 4386 children ranged from 16 to 54 years. Most newborns’ mothers (3758/4367, 86.05%) were born in Italy, followed by mothers born in Romania (101/4367, 2.31%), Albania (75/4367, 1.72%), and Morocco (60/4367, 1.37%). Concerning the newborns, 138/4386 (3.15%) were born with malformations and 352/4386 (8.03%) had a disease, most commonly neonatal respiratory distress syndrome (n=52), neonatal jaundice (n=46), and neonatal hypoglycemia (n=45).

**Conclusions:**

The NASCITA cohort is well underway and the population size will permit significant conclusions to be drawn. The key role of pediatricians in obtaining clinical data directly, along with the national-level representativity, will make the findings even more solid. In addition to promoting accurate data input, the multiple functions of the web portal, with its interactive platform, help maintain a solid relationship with the pediatricians and keep parents informed and interested in participating.

**Trial Registration:**

ClinicalTrials.gov NCT03894566; https://clinicaltrials.gov/ct2/show/NCT03894566

## Introduction

It is well known that many diseases occurring in adults can be traced back to early childhood [1,2]. In fact, nearly all domains of later health experience, including cardiovascular and respiratory disease, cognitive decline, and psychological health, have been associated with early life exposures [3]. Many different factors in childhood play a role in future health inequalities between individuals, from socioeconomic status to parental care, to lifestyle factors, but the way they are related is uncertain.

Birth cohort studies are studies that follow a group of newborns for an extended period in order to assess possible associations between exposures in early life and later health. Northern Europe has a long-lasting tradition in birth cohorts [4,5], starting from as far back as 1921 [6]. Findings from these studies have led to important knowledge in different fields, contributing to the understanding of multiple diseases and their risk factors [7,8]. These studies have also set the basis of our positive daily health behaviors. The Avon Longitudinal Study of Parents and Children (ALSPAC), for example, showed that eating oily fish during pregnancy was associated with better eye and cognitive development in children [9].

Numerous large- and small-scale birth cohorts have been set up, also in the past decade, not only in Europe but all around the world [10]. Characteristics vary greatly from one cohort to another in terms of design, objectives, size, and duration of follow-up.

Since 2003, several cohorts have also been carried out in Italy. Most of them have general aims, with data collection limited in time or to specific geographical contexts [11-19]. Italy is a special country with a public, universal health care system that should be equally accessible to all, but considerable health inequalities exist [20,21]. Up to now, no national-level birth cohort has been set up that included a large sample of the pediatric population independent of socioeconomic status or other types of limitation, such as gestational age. The Piccolipiù cohort [17], for example, recruited newborns from northern and central Italy; the NINFEA cohort [16] population was limited to women who had enough knowledge of the internet to complete online questionnaires; and the ICON cohort [19] selected preterm newborns and enrolled additional newborns of later gestational age.

In order to improve the knowledge of the health status of Italian children early on and how it is affected by social and health determinants, we set up a longitudinal, prospective, national-level, population-based birth cohort, the NASCITA study (NAscere e creSCere in ITAlia) [22]. Like many other cohorts, it addresses multiple research questions [16,17]. NASCITA is unique, however, in terms of characteristics, methodology, and population size. The findings will add important evidence, in terms of epidemiological data, for the development of specific prevention measures and interventions to improve the health status of children.

The main aim of the NASCITA cohort is to evaluate physical, cognitive, and psychological development, and health status and health resource use during the first 6 years of life in a group of newborns, and to evaluate potential associated factors.

The peculiarity of NASCITA is that data collection is designated to the general pediatricians, fitting itself into the Italian public health care system, as data reported in NASCITA are part of those routinely collected by the family pediatricians at the well-child visits. Furthermore, the data are equally distributed throughout the Italian territory.

A website and web-based system [23] were set up in order to host the cohort, provide ongoing information to pediatricians and to families, and facilitate data input on the part of the pediatricians. The system was also designed to optimize data accuracy, minimize missing data, and permit data monitoring, analysis, and reporting throughout the duration of the cohort.

This article describes the informatics methodology used to set up and maintain the NASCITA cohort with its web-based platform, and provides a general description of the participant characteristics.

## Methods

### Cohort Organization

NASCITA is embedded in Italian pediatric primary care practice. Data collection for the NASCITA cohort occurs for the most part during the 7 well-child visits planned for each child. The majority of the participating pediatricians are part of the national Pediatric Cultural Association (ACP), an association with about 2000 members consisting mainly of family pediatricians and with which the coordinating center has collaborated over the years. Participation was proposed to the ACP and forms the basis of pediatrician participation in the study. Pediatrician participation was voluntary and for free. Collaboration was also expanded through contact with other pediatric scientific societies and associations. Meetings were held during 2018 to present the study to a group of pediatricians acting as local representatives. Each representative then asked other pediatricians working in their area (at the local health unit or regional levels) to participate. Pediatrician enrollment was monitored and discussed with the local representatives. A scientific committee was set up to supervise the project, and includes professionals and lay people from different fields of expertise.

**Figure 1:**
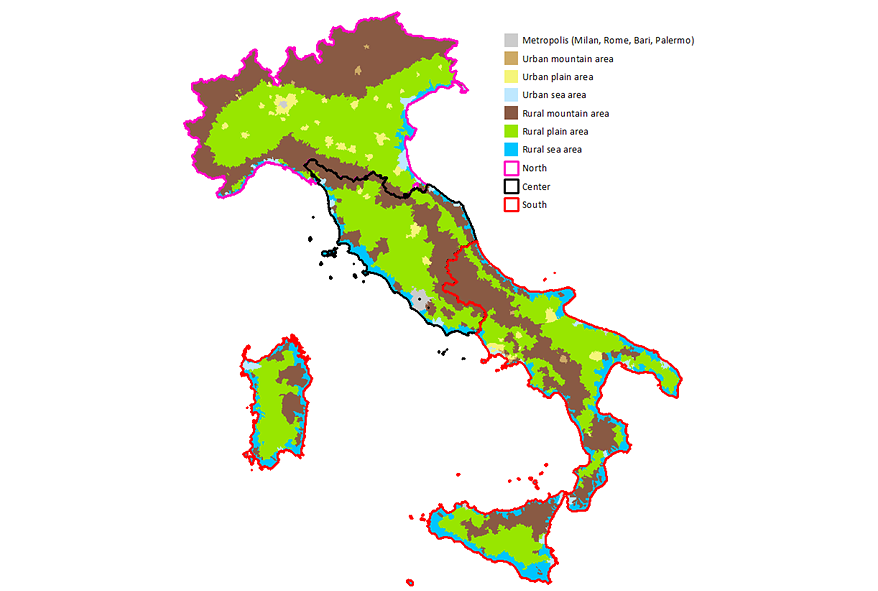
The 21 geographic clusters, identified based on ISTAT geographic and administrative criteria. ISTAT: Italian National Statistics Institute.

At the start of the study, there were 7960 cities/towns in Italy. These were classified into 21 geographic clusters (Figure 1), identified based on geographic and administrative criteria used by the Italian National Statistics Institute (ISTAT) [24]. More specifically, these take into consideration geographic area (north, center, south), setting (urban, rural), and land characteristics (plain, mountain, sea). Four cities were also selected (Milan, Rome, Bari, and Palermo), covering the different geographic areas and the islands.

Enrollment of newborns began in April 2019 and ended in July 2020. Recruitment of the newborns (and their parents) took place during the first routine well-child visit scheduled for all newborns in Italy within their first 45 days of life. All newborns assigned to the participating pediatricians were enrolled if parental consent was given. Pediatricians chose when to begin enrolling their newborns and continued to enroll for (at least) a 1-year period. Follow-up of the children will continue until at least the age of 6 years.

A minimum recruitment of 5000 newborns was calculated in order to have enough power to study common childhood exposures and outcomes.

In this article we present the characteristics of the children aged over 7 months at the time of analyses in order to provide more complete data, as pediatricians would have had time to fill in most missing data for these participants.

### Ethics

Parents were given oral and written information about the study and a consent form to sign if interested in participating. Pediatricians signed a consent form before participation as well. Withdrawal from the study was guaranteed at any time to both pediatricians and parents.

### Data Collection

Italian health care is provided free or at a nominal charge through a network of 148 local health units. The local health units assign children to a family pediatrician until they are 6 years old, after which the children can be registered with a general practitioner or remain with that pediatrician until they are 14 years old. In Italy there are about 7500 family pediatricians, for an average of 450,000 births/year [25], so about 60 newborns/year are assigned to each pediatrician. All children are scheduled 7 well-child visits at the pediatrician’s office during their first 6 years of life to ensure necessary preventive care and monitor a child’s growth and development.

Basic data are being collected and consist of data that are part of those routinely collected by the family pediatricians at the well-child visits. Some data will also be collected during each extra contact with the enrolled children. Data collection also involves parental data, such as medical history, characteristics and lifestyle, indoor and outdoor environment, and circumstances during pregnancy and around birth. Follow-up data on children will cover different fields, including physical and mental/cognitive development, nutrition and allergies, environmental exposures, and preventable infectious diseases. See Table 1 for a description of the main parts of the questionnaire. Questions were added to allow the project, in a second phase, to address specific areas such as nutrition, environment, and nurturing care.

**Table 1:** Main sections of the online questionnaires and description of the general data collected.

Section	Description
Personal data	Name, place of birth, family data such as number of family members, sibling health, parental place of birth, allergies
Medical history	Mother’s pregnancy data (including medicines, smoking and alcohol consumption, and reading out loud and listening to music), birth data (eg, newborn height, weight), perinatal medical history (eg, malformations, diseases, transfer to an intensive care unit), breastfeeding status at discharge
Visits 1-7	Medicines taken, anthropometric measures; breastfeeding status/weaning/nutrition; sleep data; age-appropriate physical examination; vitamin D + K prophylaxis; psychomotor, neurologic, and cognitive development; general health; paternal depression; language development; family habits (eg, smoking, reading out loud, listening to music, nursery school, indoor and open-air activities); home proximity to traffic or to areas of intensive farming; screen time
Extra visits	Type of contact (office, phone, home visit), diagnosis, medicines/specialist visits/examinations prescribed
Vaccination compliance	Vaccines received and adverse reactions
Exiting the cohort	Reason for exit (transfer, pulled out of study, death)

### Statistical Analysis

The analyses of the cohort data will evaluate specific research questions related to the overall aims of the study, such as the relationship between child development and the domains that affect nurturing care during the preschool period including health, nutrition, and caregiving routine; the association between the well-being of children and parental adherence to the recommendations for better child care and development; and the differences between geographical settings in educational and socialization opportunities available and in the care provided by the family pediatricians.

Data are presented as frequencies, percentages, and mean (SD) or median values. Percentages are based on denominators for which missing values have been excluded. All data management and analyses have been performed using SAS version 9.4 (SAS Institute) and ArcMap version 10.5 (ESRI). More detailed analyses will be performed, as specified in the protocol [22], and reported in future articles.

### Web Portal

**Figure 2:**
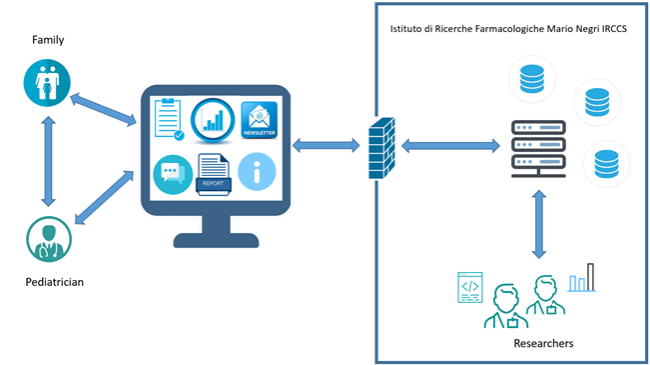
Functions of the platform and its architecture.

A specific web portal for the NASCITA cohort study was developed [23] with reserved sections for the coordinating center, registered users, and participating pediatricians. The web portal serves to assist pediatricians with data collection and to provide findings and other information during the study period to parents and pediatricians, also with the use of graphics for the analyses and data collected, based on a successful approach already reported by the coordinating center [26]. Selected sections of the portal have been translated into English. See Figure 2 for the functions of the web portal and its architecture.

**Figure 3:**
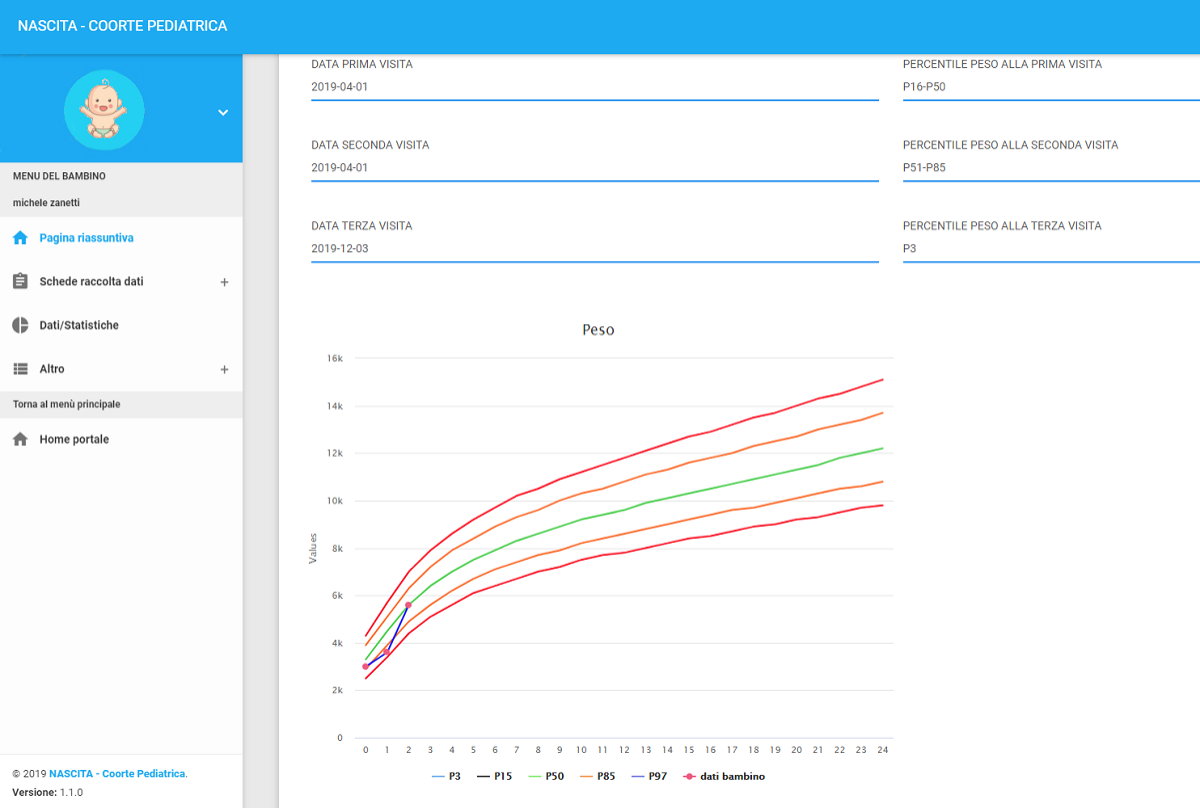
Individual child’s data plotted on growth curve, as seen by the pediatrician.

Newsletters focused on child cohorts are periodically provided in the pediatrician’s general area and contain bibliographic information of the current biomedical literature. In the private area, each participating pediatrician can access information including cohort documents; frequently asked questions; the study protocol; and pdf versions of the case report forms (CRFs); as well as patient data for input/modification; interactive data charts of his/her patients or of those of the entire cohort, including growth curves (Figure 3); and data concerning subsections of the cohort addressing areas such as nutrition and environment in which he/she participates. The pediatrician’s section on the web portal, together with individual telephone calls with the pediatricians and online and in-person meetings on the study’s progress and possible problems, serves to keep pediatricians engaged in the study.

The information for the parents section contains a growing series of cards, created in collaboration between health professionals and parents, that provide evidence-based information on the more common illnesses and problems in young children as well as answers to common questions that parents have on child care. This section also contains links to useful emergency telephone numbers and information pages.

### Electronic Case Report Form

**Figure 4:**
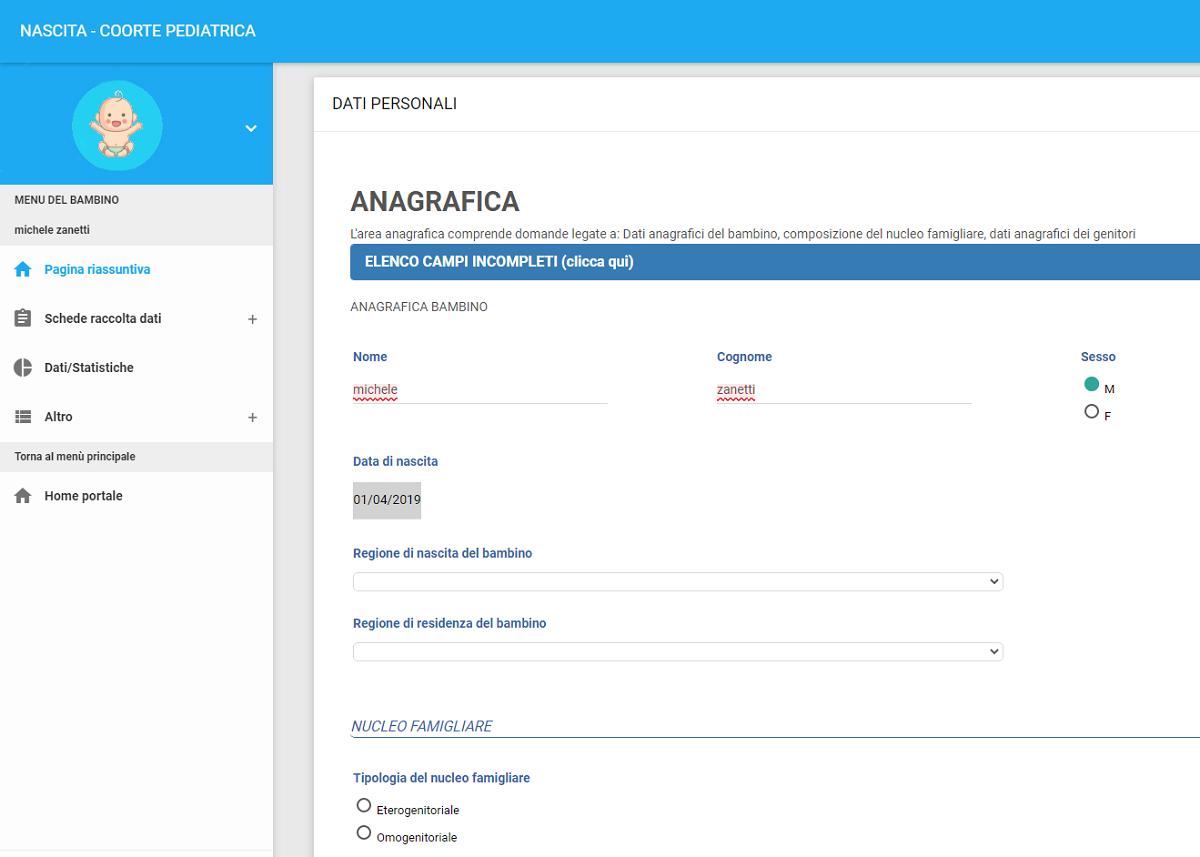
eCRF screenshots: input for second visit.

The CRF was created and tested together with a group of pediatricians. An electronic CRF (eCRF) was then set up and tested, again with the help of the pediatricians, before enrollment began. More specifically, the pediatricians were asked to register themselves and access the portal starting from January 2019 to test it. The eCRF (Figure 4) was set up in such a way as to facilitate the pediatricians’ input of data for the study and to provide fast and efficient support for any problems or doubts about data input. A “chat” section was consequently included through which pediatricians can ask for support. The eCRF includes consistency and range checks to prevent internal inconsistencies (eg, value ranges, fields with limited values, and time ranges). Data are, in any case, monitored continuously and irregularities resolved through email, chat, or phone contact with the pediatricians.

The eCRF has been structured in a way that will permit data collection to be expanded to cover the additional areas (eg, nutrition) more thoroughly in a second phase; the different data collection sections are, in fact, based on an XML definition that can easily be implemented and modified [27].

The development engine of the eCRF has been made available on Gitlab [28].

A test was performed with a group of pediatricians (including those with less experience in using the computer) to assess the additional amount of time it would take each pediatrician to enter data for a patient throughout the duration of the study. Entering data for the first follow-up visit took about 15 minutes. Multiplying this by the average number of newborns per pediatricians and, considering that after 3 months of the start of the study the subsequent regular check-ups would begin, an average of 3 hours a month in the first year was calculated, after which the amount of time necessary would decrease.

### Data Quality Control

**Figure 5:**
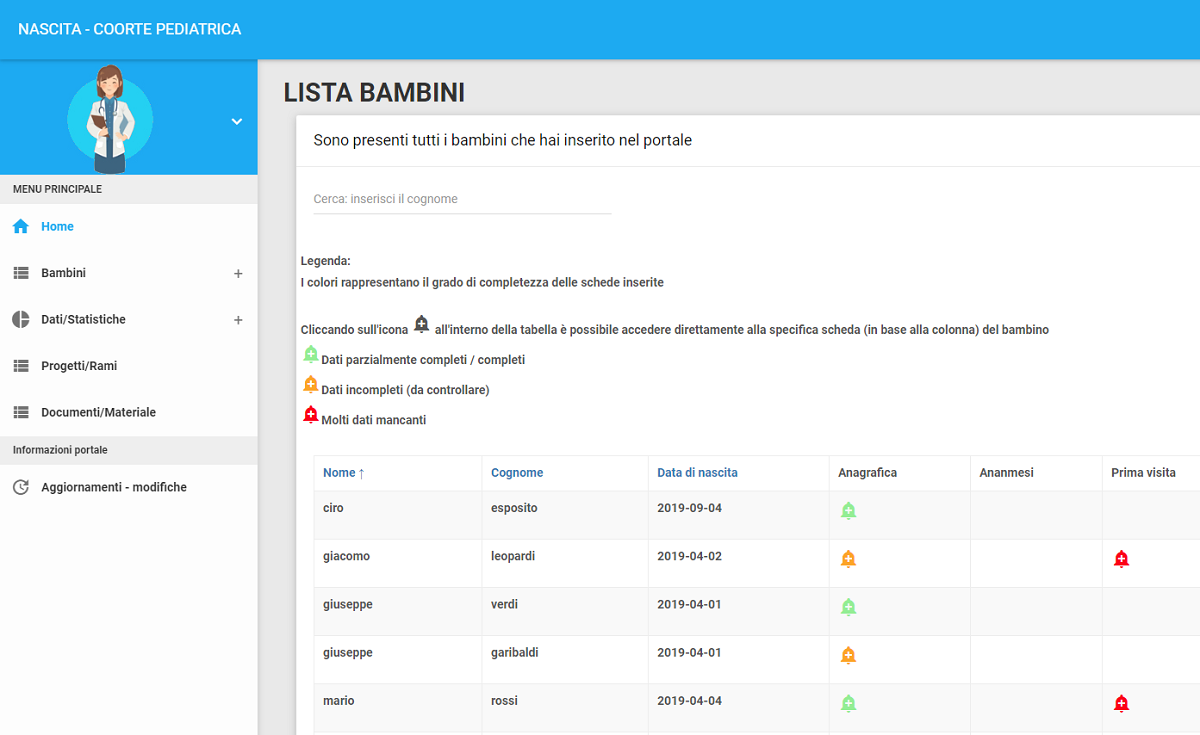
eCRF dashboard for checking completeness of data on the visits for individual patients.

A dashboard is dedicated to checking the completeness, or lack, of the visits and displays a table listing the children for whom data have been included by that pediatrician. Each column in the table represents a specific visit and shows a series of colored bells (green, yellow, or red) that indicate the completeness status of the visit (Figure 5). When a pediatrician opens the data on a specific visit, the system displays a list of the variables with missing information in order to facilitate data completion.

Frequent reports will automatically be created to monitor recruitment of pediatricians and children and the data inputted. Individual and group reports will also be created for the pediatricians and for the study’s scientific committee.

### Data Security

The private area is accessible only through authentication by pediatricians, who have been previously approved and enabled. A specific role is assigned to them, for example, *compiler*. Passwords must have at least eight characters and contain special and uppercase characters. Each pediatrician with a compiler role can insert their patients’ data. Sensitive data, such as name and last name, are encoded and visible only to the compiler. Once the data are saved, they are transmitted via secure HTTPS protocol, and are stored in databases that can be accessed only by authorized project staff (IT, statisticians). Back-ups are kept for security and disaster recovery.

## Results

Enrollment in the NASCITA cohort began on April 1, 2019, and ended on July 31, 2020. The number of participating pediatricians is 139 and the total number of children enrolled is 5166. The pediatricians are distributed throughout Italy, with 68 in the north, 29 in the center, and 42 in the south. The 5166 children enrolled represent 66.55% (5166/7763) of the children born in all of 2018 covered by these same pediatricians.

The total number of children aged over 7 months at the time of these analyses was 4386. Of these children, excluding those with missing data, 2226/4381 (50.81%) were male and 142/4370 (3.25%) were twins. The children were distributed throughout Italy, with 2025/4386 (46.17%) in the north, 882/4386 (20.11%) in the center, and 1479/4386 (33.72%) in the south. Table 2 reports the distribution of the number of these 4386 children enrolled and the percentage of children born, by cluster and geographic area, based on ISTAT data [24], and shows that there are minimal differences. The number of children enrolled per pediatrician ranged from 1 to 100 (mean 32 [SD 18.5]; median 30).

The age of the mothers at birth of the 4386 children ranged from 16 to 54 (mean 33 [SD 5.4] years; median 33 years, excluding 133 missing values), while the age of the fathers ranged from 17 to 69 (mean 36 [SD 6.3]; median 36, excluding 154 missing values). Most of the newborn’s mothers (3758/4367, 86.05%) were born in Italy; the 3 next most common countries were Romania (101/4367, 2.31%), Albania (75/4367, 1.72%), and Morocco (60/4367, 1.37%). For two-thirds of children (2892/4320, 66.94%), the mothers were married or living in civil union; for 1233/4320 children (28.54%), the mother was living with the father; and for 167/4320 (3.87%), mothers were single. Most of the children’s mothers had a university (1813/4320, 41.96%) or high-school degree (1800/4320, 41.66%), followed by a middle-school diploma (675/4320, 15.63%) and an elementary (30/4320, 0.69%) level of education. Family size (including the enrolled child) was grouped into 2, 3, 4, or >4 people, with half (2189/4311, 50.78%) of the families being made up of 3 people, followed by 1567/4311 (36.35%) made up of 4 people. Two-member families represented 1.43% (62/4311) of the total. Concerning the pregnancies, 3741/4363 (85.74%) were physiologic pregnancies, while in the remaining pregnancies gestational diabetes (203/622 mothers, 32.64%), gestational hypertension (90/622, 14.47%), and preeclampsia (41/622, 6.59%) were the most common diseases. Concerning the newborns, 139/4340 (3.20%) were born with malformations and 352/4335 (8.12%) had a disease, the 3 most common of which were neonatal respiratory distress syndrome (n=52), neonatal jaundice (n=46), and neonatal hypoglycemia (n=45).

**Table 2:** Distribution of the number of children and the number of children born in each cluster and geographic area.

Distribution/Location	Children enrolled, n (N=4386)	Population enrolled, n (%)	Births per year in Italy^a^, n (%)	Difference between the third and fourth columns, %
Metropolis: Milan	184	184/4386 (4.20)	11,267/467,640 (2.41)	1.79
Metropolis: Rome	428	428/4386 (9.76)	21,497/467,640 (4.60)	5.16
Metropolis: Bari	70	70/4386 (1.60)	2214/467,640 (0.47)	1.13
Metropolis: Palermo	62	62/4386 (1.41)	5578/467,640 (1.19)	0.22
North: urban mountain	—^b^	—	2243/467,640 (0.48)	–0.48
North: urban plain	304	304/4386 (6.93)	45,595/467,640 (9.75)	–2.82
North: urban sea	93	93/4386 (2.12)	10,258/467,640 (2.19)	0.07
North: rural mountain	228	228/4386 (5.20)	25,274/467,640 (5.40)	–0.20
North: rural plain	1151	1151/4386 (26.24)	112,824/467,640 (24.13)	2.11
North: rural sea	60	60/4386 (1.37)	5882/467,640 (1.26)	0.11
Center: urban plain	61	61/4386 (1.39)	6330/467,640 (1.35)	–0.04
Center: urban sea	1	1/4386 (0.02)	5339/467,640 (1.14)	–1.12
Center: rural mountain	90	90/4386 (2.05)	13,887/467,640 (2.97)	–0.92
Center: rural plain	233	233/4386 (5.31)	31,997/467,640 (6.84)	–1.53
Center: rural sea	68	68/4386 (1.55)	11,118/467,640 (2.38)	–0.83
South: urban mountain	8	8/4386 (0.18)	4010/467,640 (0.86)	–0.68
South: urban plain	136	136/4386 (3.10)	15,826/467,640 (3.38)	–0.28
South: urban sea	421	421/4386 (9.60)	27,281/467,640 (5.83)	3.77
South: rural mountain	98	98/4386 (2.23)	21,932/467,640 (4.69)	–2.46
South: rural plain	421	421/4386 (9.60)	47,026/467,640 (10.06)	–0.46
South: rural sea	255	255/4386 (5.81)	40,262/467,640 (8.61)	–2.80
Missing	14	—	—	—

^a^Based on data on the newborn population residing in the cluster on January 1, 2017. ISTAT demographic statistics data [24] referring to December 31, 2016, were used for clusters with missing data.

^b^—: not available.

## Discussion

### Considerations

The NASCITA cohort is based on community-level pediatric practice, involving the family pediatricians directly, as very few European cohorts do [29]. With their clinical practice, pediatricians are most in contact with patients and can promote study and action. Their involvement in child cohorts permits the collection of prospective, community-level data and allows them to contribute to optimizing both the quality of the data collected and its re-investment back into the community as health promotion interventions. In fact, pediatricians play a key role both in educating families and in implementing curative and disease prevention interventions through their routine clinical practice. They are in the optimal position to influence public health in general because adult health also depends on habits embraced when young, and pediatricians can undoubtedly influence children and their parents to adopt healthy lifestyles. In order to give something back to the pediatricians participating in the cohort, we have attempted to provide the pediatricians with useful information and interesting data, such as the interactive data charts of their patients. The system set up through which pediatricians can easily and quickly contact the cohort team for any questions or problems, and the periodic meetings organized to update pediatricians on the cohort’s status and to discuss any current issues or suggestions are additional ways to show our appreciation for all their continuing efforts. During the latest meeting we had with the participating pediatricians, online in November 2020, we described the enrolled population as it was just after enrollment closure and the next steps. On this occasion several pediatricians provided additional suggestions for improving input, resulting in the message to all that their participation and efforts are ongoing and continue to be acknowledged by the research team.

Recruitment of newborns took place over a period of 1 year for each pediatrician. This time span permitted us to avoid introducing bias related to the period of recruitment, for example, by recruiting newborns born during one season as opposed to another. The sample of children aged over 7 months reflects the distribution of births in Italy in terms of both geographic area (north, center, and south) and 21 clusters, based on the ISTAT data. Collection of data at the national level will permit the identification of differences in health care quality, such as those caused by socioeconomic inequalities present between the north and south of Italy [30], and of differences in family behaviors that influence child health status (eg, smoking or reading out loud to children). Better identifying health care–related inequalities will permit the channeling of resources where they are most needed [31]. If funding is obtained, the population enrolled could be expanded further.

As explained previously, the web portal has multiple functions and is fundamental for several reasons. The innovative aspects involve permitting the accurate input and monitoring of data through the use of a tool that creates data collection based on an xml definition, and providing pediatricians with interactive charts of current data to share with the children’s parents.

This xml-based system allows a continuous and simple updating of the CRF, saving a lot of time in the development and testing phase. In addition, saving the data in the JavaScript Object Notation (JSON) format allows greater flexibility in the database structure which, therefore, does not need to be remodified at each CRF update [32].

Furthermore, our idea for the future is to interconnect the portal with apps for parents to use to access data and to provide additional information.

### Strengths and Limitations

This is one of a very limited number of child cohorts based on the participation of family pediatricians, permitting the collection of data by those directly involved with the children and the implementation of findings to inform and help those directly involved (the children and their families). Furthermore, the large, representative population sample of newborns throughout the country, which allows stratified trends based on socioeconomic and geographic characteristics to be performed, and the use of standard measurements for anthropometric and neurocognitive parameters are among the strengths of this study.

A limit of the NASCITA cohort is that it does not collect biological samples due to the costs of data collection and storage, so it will not be able to evaluate genetic or immunological factors, for example. Resources and efforts were utilized, however, to achieve the largest population size possible in order to have enough power to study relatively common child exposures and outcomes. Another limitation is the potential bias in the pediatrician population because participation was voluntary and this may have led more motivated pediatricians to participate than others.

### Conclusions

The NASCITA cohort is well underway and its population size will permit significant conclusions to be drawn. The key role of pediatricians in obtaining clinical data directly, along with the national-level representativity, will make the findings even more solid. In addition to promoting accurate data, the multiple functions of the web portal, with its expanding, interactive platform, will help maintain a solid relationship with the pediatricians and keep parents informed and interested in participating.

## Abbreviations

ACP: Pediatric Cultural Association
CRF: case report form
eCRF: electronic case report form
ISTAT: National Statistics Institute
JSON: JavaScript Object Notation
NASCITA cohort: NAscere e creSCere in ITAlia cohort
